# *Thismia daemona* (Thismiaceae), a new species from Thailand supported by morphological and molecular evidence

**DOI:** 10.3897/phytokeys.278.202868

**Published:** 2026-08-17

**Authors:** Tosak Seelanan, Isareyakorn Chantapram, Neeranuch Taosiri, Chanachon Chuchuea, Sahut Chantanaorrapint

**Affiliations:** 1 Plants of Thailand Research Unit, Department of Botany, Faculty of Science, Chulalongkorn University, Pathumwan, Bangkok 10330, Thailand Plants of Thailand Research Unit, Department of Botany, Faculty of Science, Chulalongkorn University Bangkok Thailand; 2 PSU-Herbarium, Division of Biological Science, Faculty of Science, Prince of Songkla University, Hat Yai, Songkhla 90110, Thailand PSU-Herbarium, Division of Biological Science, Faculty of Science, Prince of Songkla University Songkhla Thailand

**Keywords:** Achlorophyllous plants, conservation status, coralliform root, endemic species

## Abstract

A new species of *Thismia* from Thailand, *T. daemona* is described based on morphological characters and molecular phylogenetic analyses. Morphological characters were examined from fresh and spirit materials using a stereo microscope. Phylogenetic analyses were conducted based on three nuclear (SSU, ITS, LSU) and two mitochondrial (*atpA*, *matR*) markers. The new species is characterized by coralliform roots, blackish flowers, reddish orange and fleshy outer tepals, inner tepals forming a mitre with three slender claviform appendages, inner surface of floral tube possessing irregular transverse bars in lower half and finely reticulations in upper half, epidermis of transverse bars and reticulations covered with white translucent trichomes, and translucent blue stamens with recurved apex. Phylogenetic analyses support its placement within *Thismia* sect. *Geomitra*, with a sister relationship to *T. betung-kerihunensis*. In addition, the new species is assessed as Critically Endangered according to the IUCN Red List categories and criteria.

## Introduction

*Thismia* Griff. is one of the largest genera of fully mycoheterotrophic plants comprising about 120 currently accepted species. It is distributed from tropical and subtropical Asia to temperate Australia and also in tropical America, with the highest number of species in Southeast Asia (Ya et al. 2024; Dančák et al. 2025; Nuraliev and Sennikov 2025; Siti-Munirah and Mohamad Alias 2025; Siti-Munirah et al. 2025). Species of *Thismia* are notoriously difficult to find due to their ephemeral nature, appearing above ground only during the flowering and fruiting season, which makes them a subject of great interest for taxonomic and ecological studies (Dančák et al. 2020). Despite recent progress in species discovery, the diversity and distribution of *Thismia* in Southeast Asia remain significantly underestimated. Thailand, with its diverse ecosystems ranging from lowland to montane forests, provides a wide range of suitable habitats for *Thismia* species. However, many remote areas of the country, particularly high-altitude habitats, remain under-explored. So far, fifteen species of *Thismia* were reported in Thailand (Larsen 1965; Chantanaorrapint and Sridith 2007, 2015; Chantanaorrapint 2008, 2012, 2018; Chantanaorrapint and Chantanaorrapint 2009; Chantanaorrapint et al. 2015, 2016, 2019; Suetsugu et al. 2017; Chantanaorrapint and Suddee 2018; Chantanaorrapint and Seelanan 2021) and discovery of additional species would be expected.

*Thismia section Geomitra* (Becc.) Kumar & S.W.Gale is one of the most interesting groups of Old World *Thismia* as it has a rather limited distribution. The section currently comprises seven species. Five of them are found in Thai-Malay Peninsula including *T. clavigera* (Becc.) F.Muell., *T. clavigeroides* Chantanaorr. & Seelanan, *T. kelantanensis* Siti-Munirah, *T. limkokthayi* Siti-Munirah & E.Chan and *T. selangorensis* Siti-Munirah & Gim Siew (Stone 1980; Chantanaorrapint and Chantanaorrapint 2009; Siti-Munirah 2018; Chantanaorrapint and Seelanan 2021; Siti-Munirah et al. 2022, 2025). Whereas *T. betung-kerihunensis* Tsukaya & H.Okada and *T. sumatrana* Suetsugu & Tsukaya occur in Borneo and Sumatra Islands, respectively (Tsukaya and Okada 2012; Suetsugu et al. 2018). The section is readily recognized by its coralliform roots, inner tepals forming a mitre with three slender claviform appendages, and stamens bearing prominent dorsal rib.

Based on morphological and molecular evidence, we herein describe a new species of *Thismia section Geomitra* discovered during recent botanical surveys in the montane evergreen forest of Thong Pha Phum National Park. This discovery further highlights the importance of protecting high-altitude forest ecosystems in Thailand as critical habitats for specialized mycoheterotrophic flora.

## Materials and methods

### Specimen collection

Two specimens of the new species of *Thismia* were collected from Thong Pha Phum National Park, Kanchanaburi Province, Thailand in 2025. In addition, material of *T. clavigeroides* Chantanaorr. & Seelanan was collected from its type locality. Voucher specimens were preserved in alcohol (70% ethanol) and deposited in BCU, BKF and PSU herbaria (herbarium acronyms according to Thiers 2026).

### Taxon sampling for phylogenetic study

**Figure 1.**
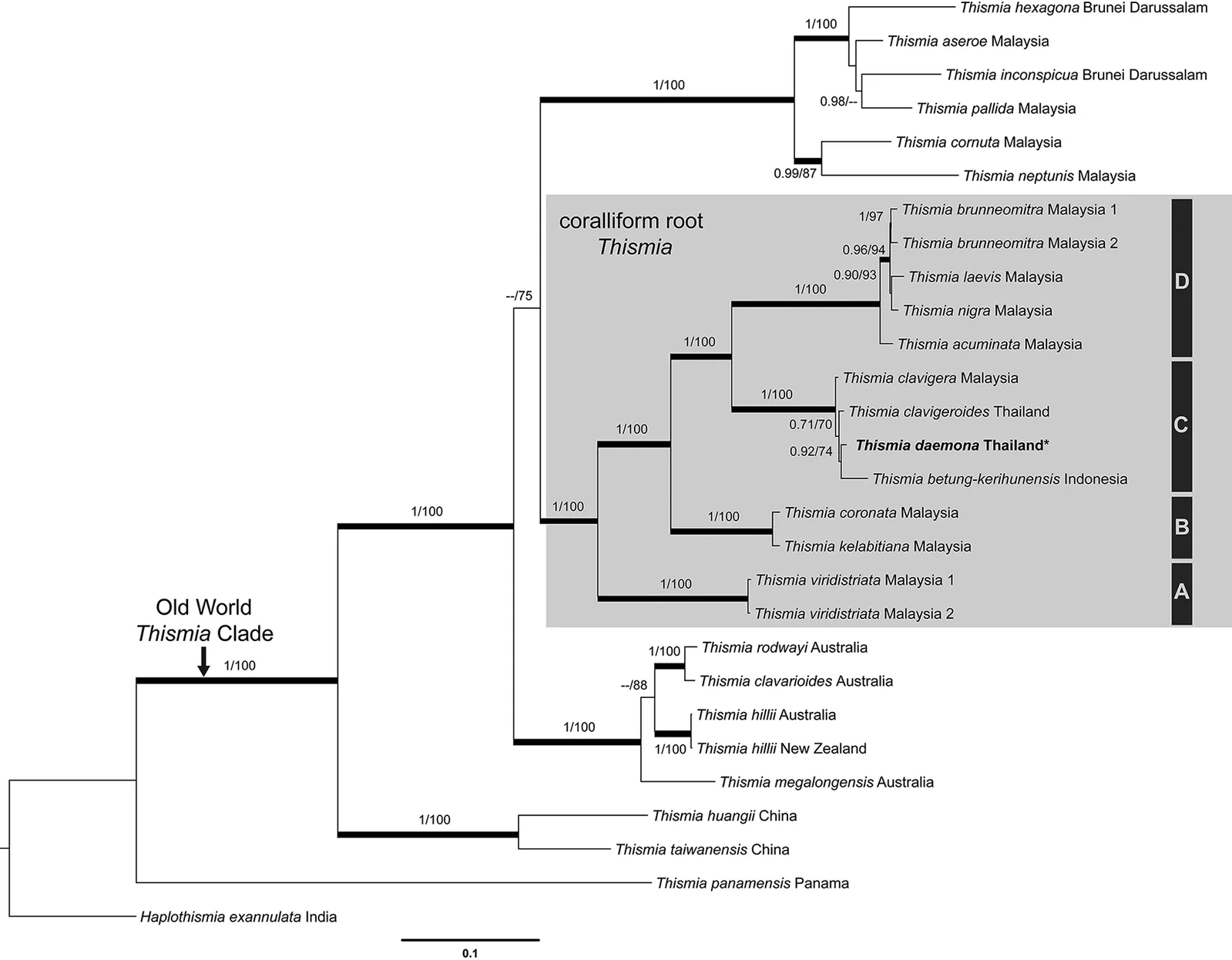
Majority-rule consensus tree based on Bayesian analysis of the combined dataset of SSU, ITS, LSU, *atpA*, *matR* markers. Bayesian posterior probabilities values (BI-PP) and ML bootstrap values (ML-BS), are indicated at branches (BI-PP/ML-BS). Thickened branches indicate BI–PP ≥ 0.95, ML–BS ≥ 80%. Support values of BI–PP < 0.70 and ML–BS < 70 are not shown or indicated by a dash (--).

One specimen of the newly described species was sequenced. *Haplothismia exannulata* Airy Shaw was chosen as the outgroup, following taxonomic relationships established in previous studies (Sochor et al. 2018). The newly generated sequences have been deposited in GenBank. Nucleotide sequences of the outgroup taxa and the remaining *Thismia* accessions were downloaded from GenBank (Fig. 1, Table 1). In total, 27 accessions representing 24 species of *Thismia* were included in the phylogenetic analyses. Detailed information regarding the studied taxa, including voucher information and GenBank accession numbers is provided in Table 1.

**Table 1.** Specimens, DNA regions and GenBank accession numbers used in the phylogenetic study. Newly generated sequences in this study in bold, a dash ‘–’ missing data.

**Species**	**Voucher information**	**Locality**	**GenBank accession numbers**				
			**SSU**	**ITS**	**LSU**	***atpA***	***matR***
*Haplothismia exannulata* Airy Shaw	*N. Sasidharan & P. Sujanapal 30476* (KFRI)	India	DQ786082	–	–	EU421037	KP420318
*Thismia acuminata* Hroneš, Dančák & Sochor	*M. Sochor et al. Bor6/17* (SAR)	Malaysia: Sarawak	MG008350	MG008338	MG008378	MG008365	MG008392
*Thismia aseroe* Becc.	*M.Y. Siti-Munirah FRI79116* (KEP)	Malaysia: Borneo	PV221322	PV230807	PV221334	PV236201	–
*Thismia betung-kerihunensis* Tsukaya & H.Okada	*H. Okada et al. HT1012* (BO)	Indonesia: Borneo	–	MK356140	–	–	–
*Thismia brunneomitra* Hroneš, Kobrlová & Dančák	*M. Sochor et al. Bor38/19* (SAR)	Malaysia: Sarawak	MN067328	MN067259	MN067290	MN067236	MN067309
*Thismia brunneomitra* Hroneš, Kobrlová & Dančák	*M. Hroneš MH2/19* (OL)	Malaysia: Sarawak	MN067322	MN067276	MN067284	MN067235	MN067301
*Thismia clavarioides* K.R.Thiele	*P. Jordan NSW 447624* (NSW)	Australia: New South Wales	KF692533	KX790903	–	KF692539	KY554924
*Thismia clavigera* F.Muell.	*L.R. Caddick 354* (K)	Malaysia: Borneo	AF309405	KY554878	–	EU421049	KY554931
***Thismia clavigeroides* Chantanaorr. & Seelanan**	***I. Chantapram & S. Chantanaorrapint 28* (PSU)**	**Thailand**	**PX959963**	**PX959961**	**PX974458**	**PX962490**	**–**
*Thismia cornuta* Hroneš, Sochor & Dančák	*M. Sochor et al. Bor2/17* (SAR)	Malaysia: Borneo	MG008353	MG008341	MG008370	MG008367	MG008384
*Thismia coronata* Hroneš, Dančák & Sochor	*M. Sochor et al. Bor11/19* (SAR)	Malaysia: Borneo	MN067320	MN067256	MN067281	MN067234	MN067300
***Thismia daemona* Seelanan & Chantanaorr**.	***N. Taosiri 345* (BKF)**	**Thailand**	**PX959964**	**PX959962**	**PX974409**	**PX962489**	**PX926481**
*Thismia hexagona* Dančák, Hroneš, Kobrlová & Sochor	*M. Sochor s.n*. (SAR)	Brunei Darussalam	KU948543	MG008342	MG008372	KU948541	MG008386
*Thismia hillii* (Cheeseman) N.Pfeiff. 1	*V. Merckx et al. NSW1_TH5* (NSW)	Australia: New South Wales	KY554861	KX790917	–	KY554871	KY554928
*Thismia hillii* (Cheeseman) N.Pfeiff. 2	*V. Merckx et al. NZ3_TH2* (NSW)	New Zealand: Pirongia	KY554862	KX790908	–	KY554872	KY554929
*Thismia huangii* P.Y.Jiang & T. H. Hsieh	*T.H. Hsieh & P.Y. Chiang 3031* (TAI)	China: Taiwan	KF692534	KY554879	–	KF692543	KY554932
*Thismia inconspicua* Sochor & Dančák	*M. Dančák & M. Hroneš MDMH2023/37* (SAR)	Brunei Darussalam	PV221316	PV230809	PV221328	PV236199	PV236212
*Thismia kelabitiana* Dančák, Hroneš & Sochor	*M. Sochor et al. Bor1/17* (SAR)	Malaysia: Sarawak	MG008355	MG008343	MG008375	MG008364	MG008389
*Thismia laevis* Sochor, Dančák & Hroneš	*M. Sochor et al. Bor9/17* (SAR)	Malaysia: Sarawak	MG008356	MG008344	MG008379	MG008366	MG008390
*Thismia megalongensis* C.A.Hunt, G.Steenbeeke & V.Merckx	*C. Hunt & G. Steenbeke 889649* (NSW)	Australia: New South Wales	KJ885661	KX790922	–	KJ885662	KY554930
*Thismia neptunis* Becc.	*M. Sochor & Z. Egertová Bor51/17* (OL)	Malaysia: Borneo	MG008357	MG008345	MG008371	MG008368	MG008383
*Thismia nigra* Dančák, Hroneš & Sochor	*M. Sochor et al. Bor46/17* (SAR)	Malaysia: Sarawak	MG008358	MG008346	MG008381	MG008362	MG008391
*Thismia pallida* Hroneš, Dančák & Rejžek	*M. Hroneš & M. Dančák Bor62/17* (SAR)	Malaysia: Sabah	MG008359	MG008347	MG008373	MG008369	MG008385
*Thismia panamensis* (Standl.) Jonker	*Aizprua 2946* (LV)	Panama	DQ786081	EU421058	–	EU421050	MT054554
*Thismia rodwayi* F.Muell.	*V. Merckx & al. TAS1_TR1* (TAS)	Australia: Tasmania	KY554865	KX790859	–	KY554875	KY554927
*Thismia taiwanensis* Sheng Z.Yang, R.M.K.Saunders & C.J.Hsu	*S.-Z. Yang et al. 28981* (PPI)	China: Taiwan	DQ786080	KY554880	–	EU421051	KY554933
*Thismia viridistriata* Sochor, Hroneš & Dančák 1	*M. Sochor et al. Bor11/17* (SAR)	Malaysia: Sarawak	MG008360	MG008348	MG008376	MG008363	MG008387
*Thismia viridistriata* Sochor, Hroneš & Dančák 2	*M. Sochor et al. Bor27/19* (SAR)	Malaysia: Sarawak	MN067326	MN067264	MN067287	MN067239	MN067306

### Morphological study

Morphological characters were studied using stereomicroscope and high-resolution macrophotography. Measurements were taken from living and spirit materials. The specimen characters were compared in detail with the protologues and relevant literature on *Thismia* sect. *Geomitra* (e.g. Beccari 1878; Jonker 1938, 1948; Stone 1980; Chantanaorrapint and Chantanaorrapint 2009; Tsukaya and Okada 2012; Siti-Munirah 2018; Suetsugu et al. 2018; Chantanaorrapint and Seelanan 2021; Siti-Munirah et al. 2022, 2025). Voucher specimens were deposited in BCU, BKF and PSU herbaria (herbarium acronyms according to Thiers 2026). The preliminary conservation assessments are based on the most recent version of the guidelines of the IUCN Standards and Petitions Committee (2024). The extent of occurrence (EOO) and area of occupancy (AOO) were calculated using GeoCAT (https://geocat.iucnredlist.org/) as outlined by Bachman et al. (2011).

### DNA extraction, amplification, and sequencing

The total genomic DNA of *T. clavigeroides* and *T. daemona* was extracted by using the Genomic DNA Mini kit (Plant) (Geneaid Biotech Ltd., Taiwan) following manufacturer’s protocols. Three nuclear markers, SSU, ITS, and LSU, and two mitochondrial markers, *atpA* and *matR*, were amplified and sequenced following Sochor et al. (2018).

The newly generated sequences were edited using Geneious Prime 2024.0.7 (https://www.geneious.com) (Kearse et al. 2012), and a consensus sequence was assembled by comparison of the forward and reverse sequence of each individual. Alignment for the consensus sequences and published sequence data retrieved from GenBank was initially performed in MEGA v.11.0.13 (Tamura et al. 2021) and then checked by visual inspection and manual adjusted in Geneious Prime 2024.0.7. Ambiguously aligned sites and gap-rich columns were excluded from phylogenetic analyses. Lacking parts of sequences and missing nucleotides were coded as missing data.

### Phylogenetic analysis

The phylogenetic trees of individual and combined datasets were constructed using Bayesian inference (BI) and maximum likelihood (ML) analysis. The individual marker sets and the combined dataset were first analyzed separately and compared for possible incongruence in the topology. As the trees showed no conflicting nodes, the datasets were combined. Bayesian inference analysis was performed using MrBayes v.3.2.7a (Ronquist et al. 2012) accessed via the CIPRES Science Gateway (Miller et al. 2010). The optimal nucleotide substitution model for each DNA region was tested in PartitionFinder2 (Lanfear et al. 2017) with the Akaike information criterion (AIC). The GTR+G model was selected for all the DNA regions except for the ITS, which was analyzed under the GTR+I+G model. The dataset was then analyzed using Markov chain Monte Carlo (MCMC) heuristic searches, performing two independent runs with four chains of 10 million generations, and trees were sampled every 3000 generations. A burn-in of 25% of the trees based on Tracer v.1.7.1 (Rambaut et al. 2018) was discarded before inferring a single majority-rule consensus tree with Bayesian posterior probability (BI-PP) confidence values. Maximum likelihood analysis was performed using IQ-Tree2 v. 2.2.2.6 (Minh et al. 2020) with a single GTR+I+G model for the concatenated dataset with 10,000 rapid bootstrapping replicates. Clades were considered supported if Bayesian posterior probability (BI-PP) was ≥ 0.90 and maximum likelihood bootstrap percentages (ML-BS) was ≥ 70% simultaneously, and sufficiently supported if BI-PP values were ≥ 0.95 and ML-BS values were ≥ 80%. Figtree (Rambaut 2017) was used to graph and edit trees.

## Results

### Phylogenetic analyses

A concatenated dataset of three nuclear (SSU, ITS, LSU) and two mitochondrial (*atpA*, *matR*) markers contained 5498 characters (1366, 1097, 891, 1099 and 1045 characters respectively). The tree topologies generated by the BI and ML analyses (Fig. 1) were congruent.

The backbone phylogeny of the genus *Thismia* is similar to that published and described by Sochor et al. (2018), Shepeleva et al. (2020) and (Ya et al. 2024). The phylogenetic reconstruction shows the monophyletic lineage of the old world *Thismia* with strong support (BI-PP = 1, ML-BS = 100). In the tree topology (Fig. 1), the coralliform root *Thismia* is divided into four clades. Clade A contains two accessions of *T. viridistriata*. Clade B consists of *T. coronata* and *T. kelabitiana*. Clade C (*Thismia sect. Geomitra*) includes *T. betung-kerihunensis*, *T. clavigera*, *T. clavigeroides* and the newly described species, *T. daemona* (which occupies a sister position to *T. betung-kerihunensis*). Clade D (*Thismia sect. Sarcosiphon* (Blume) Jonker *s.str*.) is composed of *T. acuminata*, *T. brunneomitra*, *T. laevis* and *T. nigra*.

### Taxonomic treatment

#### Thismia daemona

Taxon classification

Plantae

DioscorealesBurmanniaceae

Seelanan & Chantanaorr. sp. nov.

FB1A2FC2-8555-57EE-9ACD-526297F2451E

urn:lsid:ipni.org:names:77394258-1

Figs 2, 3

##### Type.

Thailand • Kanchanaburi: Thong Pha Phum National Park, ca. 970 m elev., 14°41.8245'N, 98°24.0630'E, 13 May 2025, *N. Taosiri 345* (holotype: BKF!; isotype: BCU!, PSU!).

**Figure 2.**
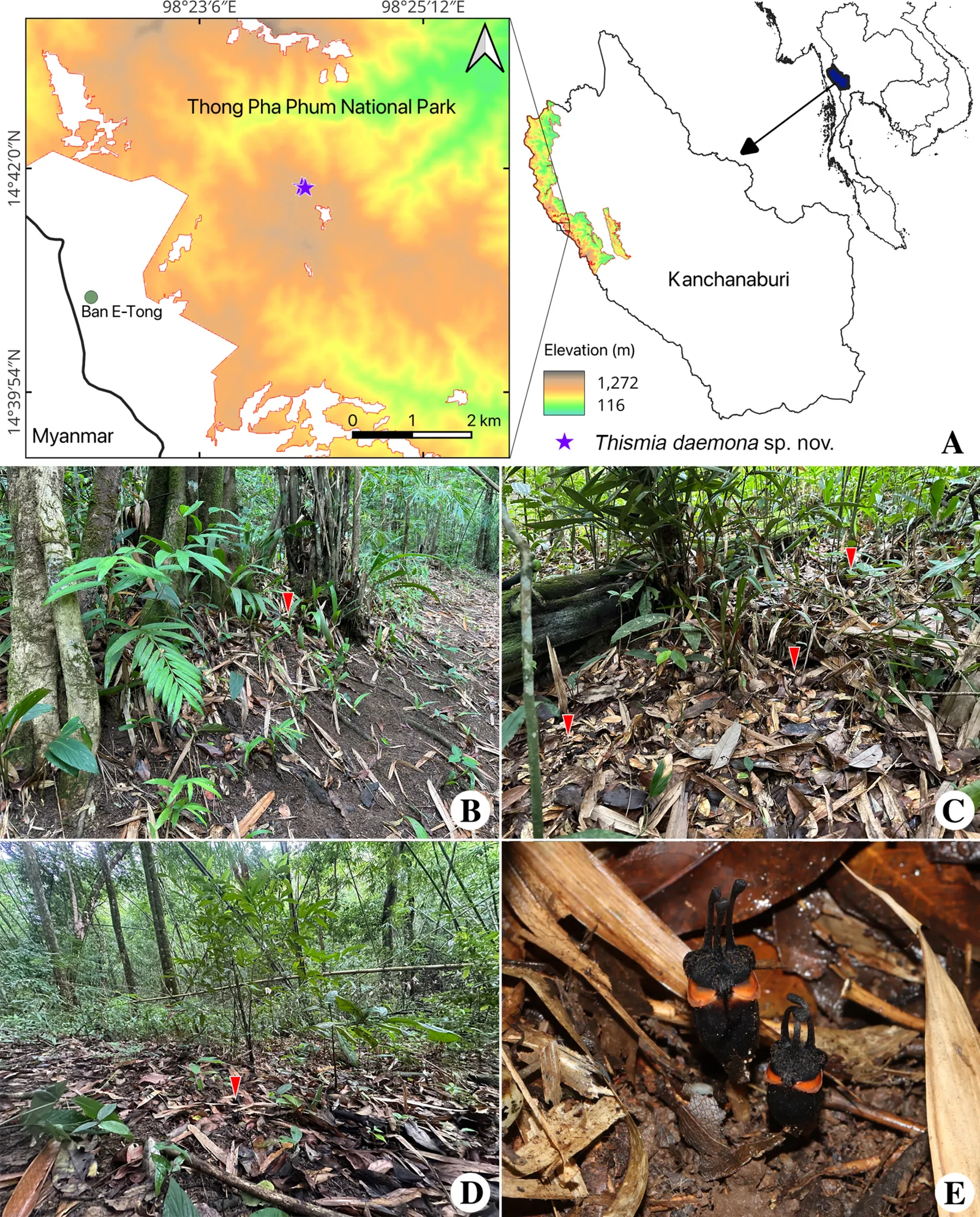
Locality and habitats of *Thismia daemona* Seelanan & Chantanaorr. **A**. Distribution map; **B–D**. Habitats; arrowheads indicate plants of *T. daemona*; **E**. Plants in natural habitat. Photographed by C. Chuchuea (**B–D**) and N. Taosiri (**E**).

**Figure 3.**
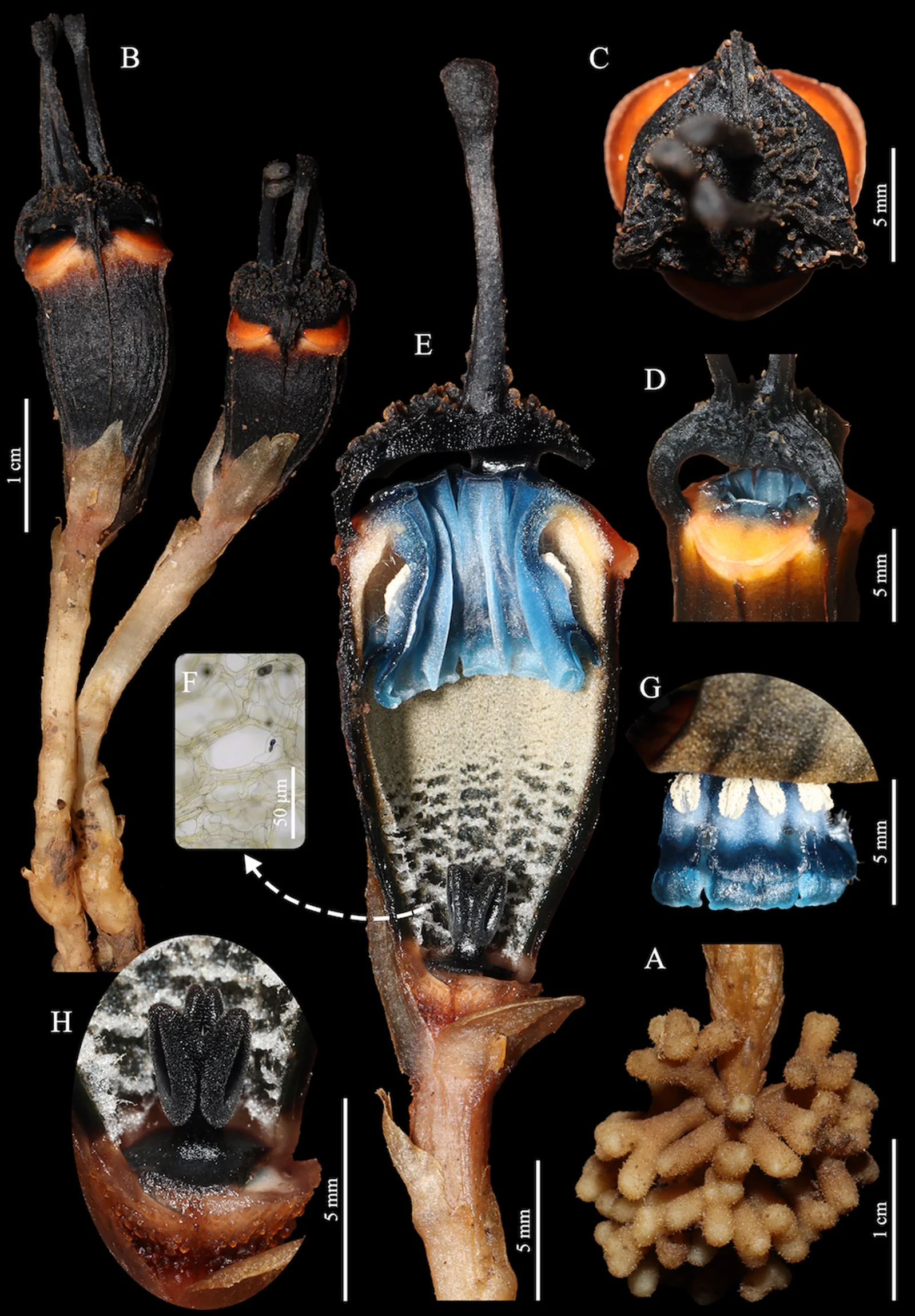
*Thismia daemona* Seelanan & Chantanaorr. **A**. Underground part showing hairy roots; **B**. Above-ground shoots with flowers; **C**. Top view of mitre showing irregular papillose surface; **D**. Side view of flower showing outer lobe; **E**. Longitudinal section of floral tube showing inner surface, three stamens, stylar column and styles; **F**. Epidermal cells of transversal bar; **G**. Stamens outer view. **H**. Stylar column and styles. Photographed by N. Taosiri (**B, C, E, G, H**) and C. Chuchuea (**A, D, F**).

##### Diagnosis.

*Thismia daemona* is somewhat similar morphologically to *T. limkokthayi*, but differs in having reddish orange and fleshy outer tepals, outer surface of mitre covered with irregular papillae, inner surface of floral tube possessing irregular transverse bars in lower half and finely reticulations in upper half, epidermis of transverse bars and reticulations covered with translucent white trichomes, translucent blue stamens, and absence of mitre foveae.

##### Description.

Achlorophyllous herb, to 125 mm tall (including flowers). ***Roots*** coralliform, pubescent, light brown. ***Stem*** 35–65 mm tall, 2.8–3.5 mm in diameter, erect, unbranched, light brown, glabrous, bearing 1–3 ﬂowers. ***Leaves*** spirally arranged, scale-like, triangular-ovate to lanceolate, increasing in size towards shoot apex, up to 8 mm long, 1–2 mm wide at base, apex acute to acuminate, pale brown, glabrous. ***Involucre bracts*** 3, similar to upper leaves but slightly larger, 10–12 mm long, 2–2.5 mm wide at the base. ***Flowers*** sessile, actinomorphic, up to 6 cm long including the ovary and appendages. ***Floral tube*** urceolate, 19–22 mm long and 7–14 mm wide, constricted above the ovary and widest in the upper part; outer surface dark brown to blackish, with 6 longitudinal ribs, glabrous; inner surface with 12 longitudinal ribs, with transverse bar in lower half and finely reticulation in upper half, epidermis of transverse bar and reticulation covered by translucent white trichomes. ***Outer tepals*** 3, free, thick, reddish orange, ca 5 mm wide at base, apex obtuse to round, surface glabrous. ***Inner tepals*** 3, blackish to dark brown, thick, cuneate, outer surface papillose, apically adnate to form a dome-shaped mitre with 3 lateral apertures and 3 hood-like accessory lobes together with 3 claviform appendages at the top; lateral aperture arch-shaped, 7.5–10.5 mm wide; hood-like accessory lobes more curved during the early stages of ﬂowering and ﬂattening when the ﬂower older and matured; claviform appendages 18–23 mm long, blackish or dark brown, papillose at base, nearly glabrous above. ***Stamens*** 6, pendent from annulus; filaments transparent blue, curved downward, with bases slightly protruding above ﬂoral tube apex, free from each other and leaving 6 apertures between them visible from above; connectives broad, bluish, fused laterally into a tube, ca. 8 mm long, each connective with conspicuous longitudinal rib along the median part of the inner surface and ending before supraconnective apex, ridge protrude and forming a sharp tooth; supraconnective free, apex bilobed, each lobe obtuse recurved inwards making the supraconnective truncate in outline, all glabrous; lateral appendage box-shaped, bright blue, not exceeding the apices of the supraconnective apex, glabrous except for sparse hairs on margins; thecae 2 in each stamen, elongated, ca. 1.5 mm long, whitish; interstaminal glands elliptic-oblong, translucent, inserted at the fusion line between the connectives. ***Ovary*** obconical, ca. 3 mm long, 4 mm in diameter, pale brown, papillose, with 6 longitudinal ribs, unilocular; placentae 3, column-like, attached to the top (roof) of the ovary; stylar column ca. 1 mm long, blackish; styles 3, blackish, oblong, ca. 2.5 mm long, bifid at the apex, with surface papillose. ***Fruit*** a cup-shaped capsule. ***Seeds*** not seen.

##### Additional specimen examined (paratype).

Thailand • Kanchanaburi: Thong Pha Phum National Park, ca. 930 m elev., 14°41.7717'N, 98°24.2317'E, 16 May 2025, *C. Chuchuea, N. Taosiri, T. Seelanan & S. Chantanaorrapint 1144* (BCU).

##### Etymology.

The specific epithet “daemona” refers to the devil-like appearance of the flower, with its dark orange outer lobes and horn-like appendages.

##### Habitat and ecology.

*Thismia daemona* was found growing amongst leaf litter, under shade of lower montane forest with small bamboo, at elevations 950–970 m.

##### Distribution.

Endemic to Thailand. Known only from its type locality at Thong Pha Phum National Park, Kanchanaburi Province; however, it may also occur in other areas in western Thailand and Myanmar with a similar vegetation type.

##### Conservation status.

*Thismia daemona* is currently known from a single population in a protected area, which is a common visiting site for tourists. Therefore, the habitat is threatened by regular tourist visits. While the EOO of *T. daemona* cannot be calculated from a single point, the AOO is formally estimated at 4 km^2^. However, the actual AOO is much smaller, as the only known population is confined to an area not larger than 0.05 km^2^. Moreover, the number of known individuals is fewer than 50. Consequently, *T. daemona* is preliminarily assigned a conservation status of CR (B2ab(iii), D) according to the guidelines of IUCN (2024).

## Discussion

*Thismia daemona* is distinguished from other species of the genus by a unique combination of morphological characters, including blackish flowers with reddish orange and succulent outer tepals. The inner tepals form a mitre with three slender claviform appendages. The outer surface of mitre is papillose. The inner surface of floral tube possesses irregular transverse bars in lower half and finely reticulations in upper half. The epidermis of transverse bars and reticulations are covered with translucent white trichomes. The stamens are translucent blue with recurved apex and each connective with conspicuous longitudinal rib ending below supraconnective apex.

Molecular phylogenetic analyses confirm that *Thismia daemona* belongs to *Thismia* sect. *Geomitra* and forms a sister relationship with *T. betung-kerihunensis*, an endemic species of Borneo, as inferred using the limited sample of DNA studied. Morphologically, *T. daemona* is easily distinguished from *T. betung-kerihunensis* by its blackish flower with reddish orange and thick outer tepals, while *T. betung-kerihunensis* has blue-green flower and thin outer tepals.

Morphological comparison indicated that *Thismia daemona* is morphologically most similar to *T. limkokthayi* from Pahang, Peninsular Malaysia in having the black to dark brown floral tube and appendages, the recurved apex of supraconnective and dark purple styles. However, the new species differs from *T. limkokthayi* by a combination of the following characters: 1) reddish orange and fleshy outer tepals (brownish orange and thin in *T. limkokthayi*), 2) outer surface of mitre covered with irregular papillae (nearly glabrous in *T. limkokthayi*), 3) inner surface of floral tube with transverse bars in lower half and irregular reticulation in upper half (absent transverse bars in *T. limkokthayi*), 4) translucent blue stamens (orange-yellowish in lower half and black/dark brown in upper half in *T. limkokthayi*), and 5) the absence of mitre foveae between the bases of claviform appendages (present in *T. limkokthayi*).

So far, there are eight species of *Thismia* sect. *Geomitra*. A complete key to the species is presented here.

### Key to species of *Thismia* sect. *Geomitra*

**Table:** 

1	Supraconnective apex truncate in outline	**2**
–	Supraconnective apex triangular in outline	**4**
2	Flowers blue green; hood like accessory lobes of mitre strongly reflexed when flower opening	***T. betung-kerihunensis***
–	Flowers blackish or dark brown; hood like accessory lobes of mitre not reflexed when flower opening	**3**
3	Inner surface of floral tube without transversal bars; outer tepal erect, thin, apex acute, thin; mitre nearly glabrous; supraconnective apex slightly exceeding the lateral appendage	***T. limkokthayi***
–	Inner surface of floral tube with transversal bars in lower part; outer tepal explanate, thick, apex obtuse to round; mitre strongly papillose; supraconnective apex distinctly exceeding the lateral appendage	***T. daemona***
4	Supraconnective apex bearing 1 triangular lobe; styles truncate	**5**
–	Supraconnective apex bearing 3 triangular lobes (the mid-lobe larger and two smaller side lobes); styles bifid	**6**
5	Mitre with 6 hood-like accessory lobes; outer tepal erect; supraconnective apex long acuminate	***T. kelantanensis***
–	Mitre with 3 hood-like accessory lobes; outer tepal reflexed; supraconnective apex acute	***T. sumatrana***
6	Mitre distinctly wider than floral tube; inner surface of floral tube with a translucent white reticulation	***T. selangorensis***
–	Mitre as wide as floral tube; inner surface of floral tube with transversal bars	**7**
7	Outer tepal erect; claviform appendages less than 1.5 cm long	***T. clavigera***
–	Outer tepal reflexed; claviform appendages 3.8–4.5 cm long	***T. clavigeroides***

## Supplementary Material

XML Treatment for Thismia daemona

## Additional information

### Artificial Intelligence (AI) use

The authors accept full responsibility for the content of the manuscript, including the disclosure of any use of AI.

No AI tools were used in the preparation of this manuscript.

### Author ORCIDs

T. Seelanan https://orcid.org/0000-0002-7768-6130

I. Chantapram https://orcid.org/0009-0001-6596-8798

N. Taosiri https://orcid.org/0009-0009-7967-1678

C. Chuchuea https://orcid.org/0009-0006-6075-4228

S. Chantanaorrapint https://orcid.org/0000-0002-9739-0994

### Data availability

All of the data that support the findings of this study are available in the main text.
